# Multi-Target Inhibition of F10/F2/PAR1 Through In Silico Drug Repurposing of Avodart and Naldemedine to Prevent Thrombotic-Induced Sudden Cardiac Arrest

**DOI:** 10.3390/biomedicines14051120

**Published:** 2026-05-15

**Authors:** Abeer M. Al-Subaie, Sayed AbdulAzeez

**Affiliations:** 1 Department of Clinical Laboratory Sciences, College of Applied Medical Sciences, Imam Abdulrahman Bin Faisal University, Dammam 31411, Saudi Arabia; 2 Department of Genetic Research, Institute for Research and Medical Consultations (IRMC), Imam Abdulrahman Bin Faisal University, Dammam 31441, Saudi Arabia

**Keywords:** thrombotic disorders, drug repurposing, coagulation cascade, virtual screening, multi-target antithrombotics, MD simulations, Factor X inhibition, Naldemedine

## Abstract

**Background:** Thrombotic disorders remain one of the leading causes of global mortality, necessitating the discovery of anticoagulants with broader therapeutic windows and multi-target efficacy. This study aimed to identify FDA-approved drugs capable of simultaneously inhibiting three critical nodes of the coagulation cascade: Factor X (F10), Proteinase-activated receptor 1 (PAR1) and Prothrombin (F2). **Methods:** High-confidence 3D structures of coagulation cascade proteins were established using AlphaFold2 and validated via MolProbity (Favored regions > 91%). A library of 1657 compounds from the Zinc database was screened using PyRx, followed by rigorous ADMET profiling to evaluate pharmacokinetic viability. The structural integrity and binding kinetics of the top candidate drugs were further analyzed through Molecular Dynamics simulation for 100 ns. **Results:** Virtual screening and downstream analysis identified 30 multi-target drugs. Avodart and Naldemedine were observed to have superior pharmacokinetic equilibrium. Compared to the other two drugs (Digoxin and Ledipasvir), Avodart and Naldemedine showed high affinity, higher adherence to drug likeness, lower metabolic inhibition risks and lack of acute toxicity, and were therefore the most suitable candidates. The 100 ns MD simulations revealed Avodart and Naldemedine to have the highest level of interaction stability and favorable MM-GBSA energies with Factor X, whereas Ledipasvir and Digoxin exhibited significant structural instability. **Conclusions:** The study proposes Avodart and Naldemedine as promising candidates for drug repurposing in antithrombotic therapy. This study provides a computational blueprint for the development of next-generation, broad-spectrum anticoagulants.

## 1. Introduction

Coronary thrombosis and sudden death affect 300,000 to 400,000 people under 35 annually in the USA [1]. Thrombotic disorders, including deep vein thrombosis, pulmonary embolism, myocardial infarction and stroke, remain a leading cause of morbidity and mortality worldwide [2]. According to estimates, 1 in 4 deaths globally in 2010 were caused by thromboembolic disorders [2]. The coagulation cascade represents a complex series of proteolytic reactions essential for hemostasis, yet its dysregulation is central to pathological thrombosis [3]. Key enzymatic factors within this cascade are well-established targets for antithrombotic therapy. Specifically, Thrombin (Factor II, F2) and Factor X (F10) are central proteases responsible for the amplification and propagation of clot formation. Thrombin in particular not only converts fibrinogen to fibrin but also activates platelets via the protease-activated receptor 1 (PAR-1, F2R), cementing its role as a multi-functional therapeutic target. Furthermore, Prothrombin, the zymogen precursor to Thrombin, offers an upstream point of intervention. The continuous need for improved antithrombotic agents with enhanced efficacy and reduced bleeding risk necessitates rigorous investigation into novel inhibitory scaffolds.

Thrombosis functions as a pathological signaling trigger. Proteinase-Activated Receptors (PARs) are activated by Factor Xa and Thrombin in addition to simple clotting, which promotes inflammation and the advancement of disease. Multi-target inhibition is therefore essential for both anticoagulation and atherosclerotic stabilization because thrombosis promotes plaque instability leading to atherothrombosis [4].

### In Silico Screening and Drug Repurposing

Conventional drug discovery is frequently prohibitively costly and time-consuming. As a result, computational techniques, molecular docking and virtual screening in particular, have developed into essential resources for quickly and affordably locating viable therapeutic candidates [5]. Virtual screening enables the high-throughput assessment of extensive chemical libraries against designated protein targets, significantly diminishing the quantity of compounds necessitating costly in vitro and in vivo testing. Additionally, drug repurposing, which involves finding new therapeutic uses for already-FDA-approved drugs, greatly reduces the time and money needed for preclinical development. The PyRx platform, which incorporates the AutoDock Vina engine, is a widely adopted molecular modelling tool for performing rapid and accurate virtual screening campaigns against known protein binding sites.

The current study objective is to evaluate the binding affinities of a large library of FDA-approved compounds against three crucial protein targets essential to the coagulation pathway—Factor Xa (F10), PAR-1 (F2R) and Prothrombin—using PyRx virtual screening [6], building on the potential of computational drug repurposing. Our main goal was to find and characterize new multi-target inhibitors with favorable predicted binding energy and molecular interactions. In order to find safer and more effective treatments for thrombotic diseases, the compounds exhibiting the strongest theoretical efficacy across these targets will be given priority as promising antithrombotic leads for upcoming in vitro and in vivo validation.

## 2. Materials and Methods

**Figure 1:**
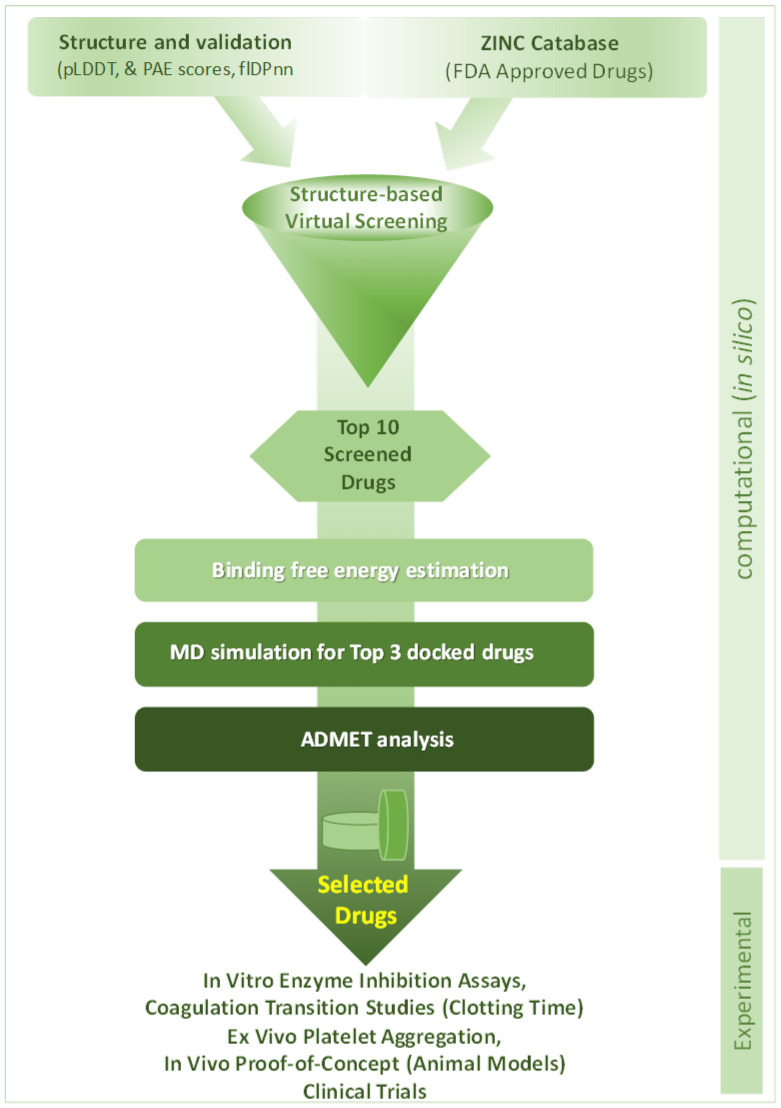
Integrated in silico and experimental pipeline for computational drug repurposing and targeting of the coagulation cascade towards antithrombotic drug development and discovery.

This study used a complete computational workflow with multiple specialized software tools (Figure 1). Discovery Studio Visualizer 2020 and PyMOL 3.0 [7,8] were used to prepare the proteins and visualize their interactions. PyRx 1.16 was used for high-throughput virtual screening. AutoDock4 was used for molecular docking, and AMBER was used to run molecular dynamics simulations to confirm the hit compounds. The pharmacokinetic and toxicological properties were analyzed using ADMET analysis with SwissADME ADMETlab 3.0 and pkCSMv1.0 [9,10,11].

### 2.1. Protein Structure Retrieval and Active Site Retrieval from Pictorial Database of 3D Structures

The three-dimensional structures of the high-resolution X-ray crystal structures (FXa PDB ID: 2P16, PAR1-PDB ID: 3VW7, Prothrombin PDB ID: 3NXP) were retrieved from the RCSB Protein Data Bank.

The active site is the specific pocket in which the chemical reaction occurs and the substrate binds. In contrast, an allosteric site is a separate, distant regulatory location. When a molecule binds to active site it changes the enzyme shape, effectively switching its activity on or off away from the main site. The active sites of the targets of F2, F10, and F2R proteins were retrieved using the PDBsum database. The PDBsum is a database with ligand protein interaction information of those structures deposited in PDB. The PDBsum gives information about the non-bonded and hydrogen-bonded interactions of the ligand with the protein, The target proteins are FXa, PDB ID: 2P16, PAR1-PDB ID: 3VW7, and Prothrombin PDB ID: 3NXP and were searched in PDBsum. The active sites for the target protein and the hydrogen-bonded interacting residues along with the non-bonded interactions were retrieved. Hence, all proteins were used for the docking process after trimming the structure and deleting all the water molecules and heteroatoms.

### 2.2. FDA-Approved Drugs

The ZINC 20 database [12] (Irwin and Shoichet Laboratories, San Francisco, CA, USA) is a curated database of 230 million compounds, including FDA-approved drugs, for virtual screening. Every medication from the ZINC database that has received FDA approval was obtained. The National Library of Medicine runs the publicly available chemical database PubChem. It includes detailed chemical structures of 1632 drugs, with characteristics and outcomes of biological assays.

### 2.3. Protein Preparation

The proteins were prepared for molecular docking using the Dock Prep module in UCSF Chimera (v1.14). Initial processing involved the removal of the co-crystallized inhibitor, structural water molecules and non-catalytic chains to isolate the primary receptor domain. Missing side chains were reconstructed using the Dunbrack rotamer library. To facilitate accurate electrostatic calculations, polar hydrogen atoms were added and partial atomic charges were assigned using the Gasteiger charge method. Finally, the structure underwent a brief energy minimization (100 steps of steepest descent and 10 steps of conjugate gradient) to resolve local steric clashes and stabilize the grid generation.

### 2.4. Ligand Preparation

Approximately 1657 medicinal molecules in SDF format were downloaded from the ZINC20 database with the “world” filter; the database contains approved pharmaceuticals in significant jurisdictions such as the FDA. The ligands were put into the PyRx dashboard, and OpenBabel 2.3.0 was used to minimize energy and convert them to pdbqt format.

### 2.5. Virtual Screening

Virtual screening was performed using the PyRx 0.9.9 (academic license) software package [6,13,14], which utilizes the AutoDock Vina engine. A library of 1657 FDA-approved drugs was screened against three key coagulation pathway receptors: Factor X (FX), Protease-Activated Receptor-1 (PAR-1/F2R) and Prothrombin. Virtual screening was used to discover possible inhibitors of Thrombin. The X-ray crystal structures of proteins were downloaded and used for virtual screening. The PDB structures of F2, F10, F2R and the ZINC database containing FDA-approved medicinal compounds were loaded into the PyRx workspace. Compounds from the ZINC database were screened against possible drug targets using PyRx 1.1, used for computational drug discovery with the PyRx 1.1 virtual screening tool. Three-dimensional structures of all compounds were created, and Open Babel in PyRx was used to reduce their energies, utilizing the Universal Force Field (UFF). Each FDA-approved medication molecule was virtually screened against F2, F10 and F2R proteins separately. Docking was carried out using a grid box containing the active site of each receptor at the center_x = −4.7, center_y = −29.0, center_z = 19.8, set size_x, y, z = 25. Compounds with the best (most negative) scores were chosen for additional research after the binding affinities (kcal/mol) were examined. To run screening, we used the cloud-based supercomputer at the Imam Abdulrahman Bin Faisal University. To validate the in silico docking protocol, validation in silico docking was carried out using Factor Xa with known inhibitors (Apixaban and Rivaroxaban) using both PDB and AlphaFold structures (Figure S1).

### 2.6. MD Simulation

The systems were parameterized using the OPLS-2005 forcefield [15,16] and solvated with the explicit SPC water model. An orthorhombic periodic boundary box was defined with a 10 Å buffer distance from the protein in all directions. To maintain electrical neutrality, appropriate Na^+^ counterions were added and NaCl was introduced to achieve a physiological salt concentration of 0.15 M [17]. Molecular dynamics simulations were used to better understand the behavior of the protein–ligand complex of FX with selected ligands in an aqueous environment. We used Desmond software (version 4.1) (academic license, D.E. Shaw Research) to simulate the optimal docked conformation for the target protein [7]. In addition to the system built using the System Builder tool, the Preparation Wizard included optimization, minimization and filling in of any gaps [18]. The simulation utilized a TIP3P water model with a cubic box and 10 Å side length. To ensure electrical neutrality, the net charge of the complex was neutralized. The simulations were carried out in the NPT ensemble, simulating constant pressure (1 bar) and temperature (300 K) with the Berendsen coupling method, and obtained the final poses for the interacting four complexes at the end. A 100 ns production simulation was completed for the protein–ligand complex after a 100 ns equilibration phase.

The molecular mechanics/generalized Born surface area (MM-GBSA) method was used to calculate the binding free energy of the protein–ligand complexes. The Python 3.8 script thermal_mmgbsa.py was used to carry out the computations on the MD simulation trajectory using the OPLS-2005 forcefield and the VSGB implicit solvation model. The final 50 frames of the trajectory were chosen for study at one-frame sampling intervals. Using the additivity principle, the individual energetic contributions were added up to determine the Prime MM-GBSA binding free energy (kcal/mol). Coulombic, covalent, hydrogen bonding, van der Waals, self-contact, lipophilic, and solvation energy components related to the protein and the ligand made up the overall binding energy.

The following formula was used to determine Δ*G_bind_*:

$${\Delta G}_{bind}={\Delta G}_{MM}+{\Delta G}_{Solv}-{\Delta G}_{SA}$$

The binding free energy of the system is denoted by Δ*G_bind_* in this equation. The difference in molecular mechanics energy between the protein–ligand complex and the total of the unbound receptor and ligand energies is described by Δ*G_MM_*. The difference in solvation free energy between the complex and the individual receptor and ligand is represented by Δ*G_solv_*. The change in surface area-related energy following complex formation is represented by Δ*G_SA_* [19].

## 3. Results

### 3.1. Structural Evaluation

To ensure the structural integrity and conformational accuracy of the targets used in this study, both experimental crystal structures and computationally predicted models were evaluated. Three-dimensional (3D) coordinates for the three target proteins (FXa PDB ID: 2P16; PAR1-PDB ID: 3VW7; and Prothrombin PDB ID: 3NXP) were retrieved from the Protein Data Bank as gold-standard experimental structures for molecular docking. Simultaneously, the corresponding primary sequences were used to generate de novo models via AlphaFold2. pLDDT and PAE measures were used to evaluate the predicted models from the Alphafold of Coagulation Factor X (F10), Proteinase-activated receptor 1 (PAR1) and Prothrombin. pLDDT score ranges from 0 to 100 were considered to assess the agreement between predicted and experimental Factor X (F10), PAR-1 (F2R) and Prothrombin (F2) structures. Lower values indicate a higher risk of intrinsic abnormality [19,20]. The predicted model of Factor X has an average pLDDT value of 80.25, PAR-1 has an average pLDDT value of 74.56, and Prothrombin has an average pLDDT value of 83.94, indicating overall high confidence, with specific regions exceeding 90 (Supplementary Figure S2). MolProbity was used to validate and analyze molecular 3D structures by assessing factors like geometry, steric clashes and overall structural validity [21]. MolProbity analysis of Factor X (F10) revealed that 91.2% (443/486) of all residues were in favored (98%) regions and 98.4% (478/486) of all residues were in allowed regions. PAR-1 (F2R) identified with 91.0% (564/620) of all residues in favored regions, and 95.8% (594/620) of residues were in allowed regions. MolProbity analysis of prothrombin revealed that 91.0% (564/620) of residues were in favored regions and 95.8% (594/620) of residues were in allowed regions of the Ramachandran plot [22].

### 3.2. Virtual Screening of F10/F2/PAR1 

**Figure 2:**
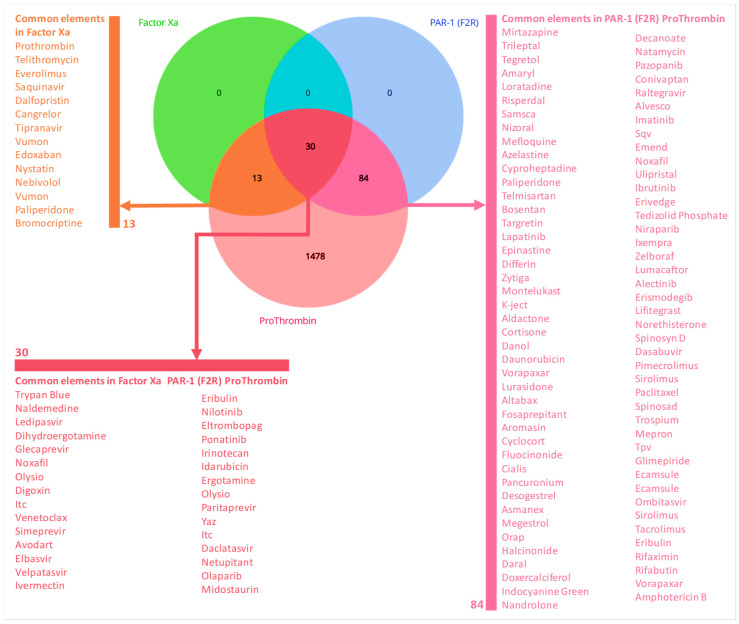
Venn diagram of virtual screening results across coagulation targets. This figure illustrates the overlap of potential drug candidates identified through structure-based virtual screening against three key proteins in the coagulation cascade. The screening was conducted on Factor X (F10), Proteinase-activated receptor 1 and Prothrombin. The central intersection highlighting the 30 common elements includes prioritized candidates for molecular dynamics simulation to determine their viability as broad-spectrum antithrombotics.

A database of 1657 approved drug molecules was screened against Factor X, PAR-1 and Prothrombin using the PyRx 0.9.9 (academic license) software package from vina docking results. A total of 30 compounds were identified as common hits across all three targets (Factor X, PAR-1 and Prothrombin) (Table 1; Figure 2). These represent the primary multi-target leads for further development. A total of 13 compounds showed specific affinity for both Factor X and Prothrombin, while 84 compounds were common to PAR-1 and Prothrombin. Prothrombin yielded the highest number of unique hits (1478 compounds), whereas Factor Xa and PAR-1 showed no unique hits within this specific filtered set (Figure 2).

**Figure 3:**
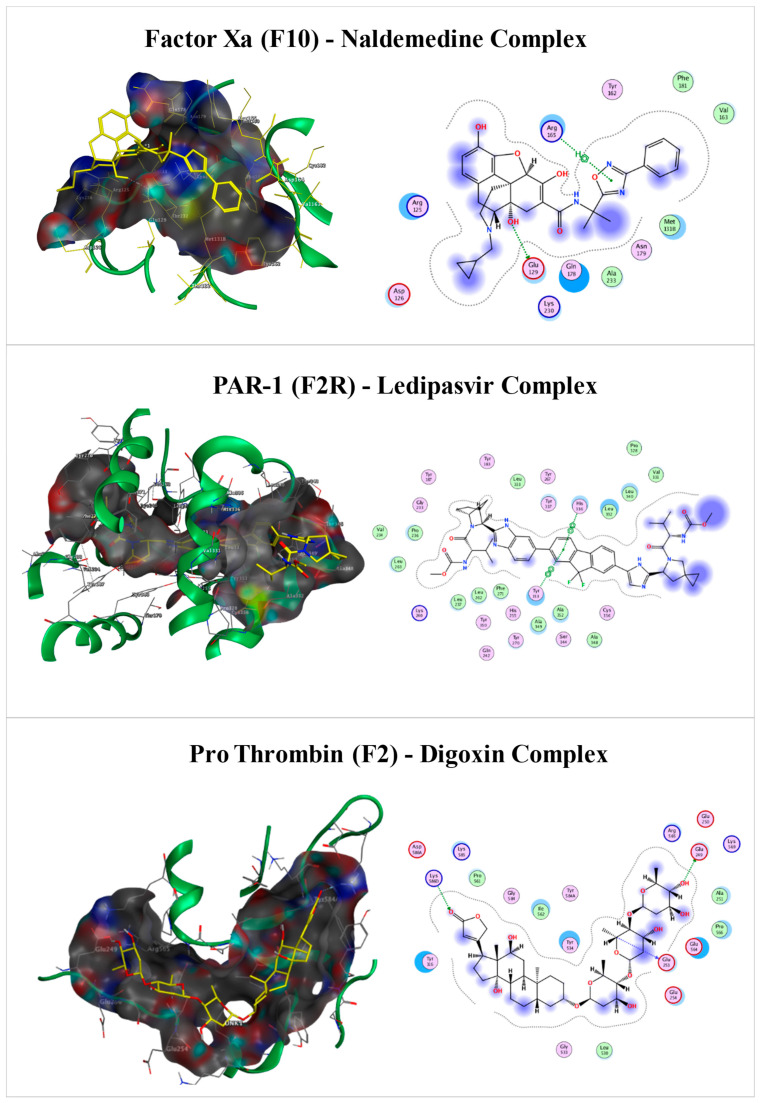
Molecular docking analysis of selected compounds with key coagulation targets. This figure illustrates the binding interactions and structural orientations of the selected ligands identified through virtual screening against three targets (Factor X, PAR-1 and Prothrombin) in the coagulation cascade. * indicates the atom involved in a hydrogen-bonding interaction.

The integrated in silico screening of the FDA-approved ZINC database against three coagulation targets, Factor X (F10), Proteinase-activated receptor 1 (PAR1) and Prothrombin (F2), revealed significant multi-target drugs (Table 1). Initial structural validation using pLDDT and IDDT plots confirmed high-confidence folds for the target proteins, particularly within the catalytic domains essential for ligand binding (Figure S2). Through structure-based virtual screening, 30 common FDA drug elements were shortlisted that were observed to have high binding affinities across all three targets (Factor Xa, PAR-1 and Prothrombin). Among these, Digoxin (zinc000242548690) showed the highest individual affinity, reaching −10.4 kcal/mol against prothrombin and being stabilized by hydrogen bonds and salt bridges. Ledipasvir (zinc000150338819) showed the most balanced multi-target profile, with an average affinity of −8.93 kcal/mol observed with strong contacts within the PAR1 orthosteric pocket. Naldemedine (zinc000100378061) also emerged as a top drug with high geometric complementarity within the Factor X active site through a hydrogen bond (Figure 3).

The reliability of the search algorithm was confirmed by validation in silico docking using FXa with known inhibitors Apixaban and Rivaroxaban. The successful replication of native binding with an RMSD of 0.000 Å (Apixaban) and 0.190 Å (Rivaroxaban) confirms the reliability of our search algorithm and forcefield (Supplementary Figure S2).

### 3.3. ADMET Analysis

The ADMET analysis revealed that both Naldemedine and Avodart are the most promising FDA drug candidates compared to Digoxin and Ledipasvir (Table 2). Naldemedine exhibited a relatively balanced profile, with a molecular weight of 570.25 g/mol. However, its bioavailability probability (F 50% = 1.0) suggested a high probability of low bioavailability (<50%). A lack of eye corrosion and moderate synthetic complexity (SAscore = 5.0) were observed in Naldemedine. Avodart was identified with specific drug-likeness parameters, although it deviated from the GSK rules due to its molecular weight (528.22 g/mol) exceeding 400 and its being on the edge of the Golden Triangle. Avodart has high lipophilicity (log *p* = 5.596) and very high plasma protein binding (98.3%). The comparative analysis revealed that the other two drugs selected, Digoxin and Ledipasvir, have notable pharmacokinetic hurdles. Digoxin was identified with a high molecular weight (780.43 g/mol) and high TPSA (203.06), resulting in poor drug likeness (QED = 0.162). ADMET analysis revealed that Ledipasvir acts as a potent inhibitor for specific enzymes (probability = 1.0), which increases drug–drug interaction risks, though it is a non-inhibitor for CYP3A4. The analysis also identified the anticoagulant Rivaroxaban with an optimal log *p* value of 1.601 and moderate TPSA. Overall, Naldemedine remains a good choice among the four selected drugs followed by Avodart, even though both selected drugs have limitations regarding specific toxicities.

### 3.4. MD Simulation of Protein–Ligand Complexes

**Figure 4:**
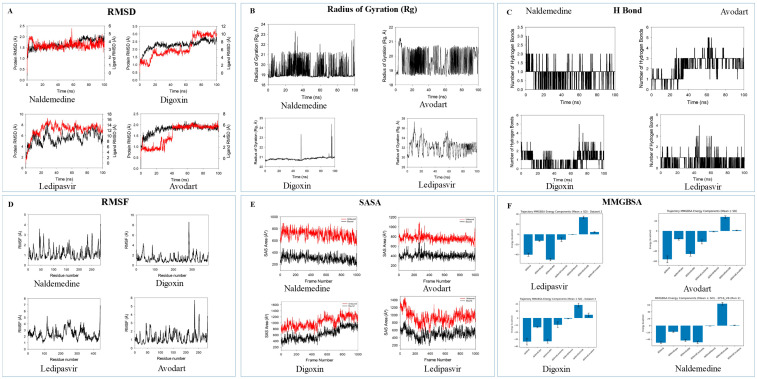
Molecular dynamics (MD) simulation analysis of Naldemedine, Digoxin, Ledipasvir and Avodart in complex with Factor X. The 100 ns trajectory analysis of the four complexes identifies the structural stability, compactness and binding energies of the four drug–Factor X complexes. (**A**) Time-dependent RMSD profiles of Factor X backbone and the selected drug. Naldemedine and Avodart display minimal fluctuations, indicating stable binding with Factor X. Ledipasvir exhibits more deviations. Black: protein (left axis); red: ligand (right axis). (**B**) Radius of gyration of Factor X compactness throughout the 100 ns. Digoxin and Avodart complexes maintain a highly consistent radius of gyration. Naldemedine displays fluctuations. (**C**) Hydrogen bond analysis between the selected drug and the active site residues of Factor X. Avodart with a consistent network of hydrogen bonds, showing high binding affinity. (**D**) RMSF results of four drug–Factor X complexes. Naldemedine and Avodart complexes show ligand-induced stabilization. (**E**) Solvent-accessible surface area profiles for four drug–Factor X complexes. The SASA values show that the Avodart and Naldemedine complexes remain shielded from the solvent upon ligand binding. (**F**) MM-GBSA binding free energy calculations of four drug–Factor X complexes. All four compounds exhibit favorable (negative) values to the complex stability.

The 100 ns molecular dynamics simulation analysis of the selected drugs—Naldemedine, Digoxin, Ledipasvir and Avodart—in complex with Factor X highlighted Avodart and Naldemedine as the most promising candidates for the future experimental studies (Figure 4). The molecular dynamics simulation analysis revealed superior structural stability and binding affinity for compounds such as Avodart and Naldemedine compared to the other two ligands, Digoxin and Ledipasvir. Avodart (ZINC3932831) exhibited the highest level of interaction stability and was characterized by a highly stable RMSD profile. In addition, MD simulation analysis revealed a hydrogen bond network maintaining 2–5 bonds throughout the analysis. Furthermore, Naldemedine (ZINC100378061) showed a slightly lower hydrogen bond than Avodart and maintained structural compactness (radius of gyration) and a stable solvent-accessible surface area (SASA) throughout the MD simulation analysis. These observations clearly indicate that the binding ability of Naldemedine is likely stabilized by hydrophobic interactions within Factor X. In contrast, the other two selected drugs, Ledipasvir and Digoxin, were noted to have structural fluctuations and deviations in protein compactness. These observations regarding Ledipasvir and Digoxin during MD simulation analysis suggest that both drugs are unfavorable for further analysis and future experimental studies. MM-GBSA-based thermodynamic analysis confirmed that both Avodart and Naldemedine exhibited favorable total binding free energies. Therefore, due to the high stability and favorable energetic profiles observed, Avodart and Naldemedine were chosen as the candidate drugs for further experimental validation and optimization studies.

## 4. Discussion

Thrombotic diseases remain a primary cause of death worldwide and their treatment requires the development of anticoagulants with broad therapeutic and multi-target efficacy. According to estimates, one in every four deaths worldwide in 2010 was caused by disorders associated with blood clots. A concept shift from conventional anticoagulation to multi-target strategies is required for the clinical management of thrombotic disorders, especially those that result in sudden cardiac arrest. Recognizing the importance of drug repurposing, this study used a computational methodology to assess numerous FDA-approved drugs as potential candidates against thrombotic diseases. In order to find FDA-approved medications that can concurrently modulate three crucial nodes of the coagulation cascade—Factor X, Prothrombin, and Proteinase-activated receptor 1this study used an integrated in silico pipeline. By focusing on this triple target, we anticipated being able to identify a therapeutic lead that addresses the underlying inflammatory triggers to prevent mechanical clot formation [23].

To ensure the structural integrity and conformational accuracy of the targets used in this study, both experimental crystal structures and computationally predicted models were evaluated. Three-dimensional (3D) coordinates for the three target proteins were retrieved from the Protein Data Bank (PDB) as gold-standard experimental structures for molecular docking. Simultaneously, the corresponding primary sequences were utilized to generate de novo models via AlphaFold2. The use of AlphaFold2 provided high-confidence structural templates, evidenced by pLDDT scores [11] exceeding 80 for all three targets. MolProbity analysis [21], which showed that more than 91% of residues fell within the preferred regions of the Ramachandran plot, further supported the structural validity. These measures are essential because they verify that the orthosteric pockets and catalytic domains used for docking were reflective of bioactive states and structurally correct. The virtual screening landscape and lead selection for virtual screening of 1657 FDA-approved compounds revealed a significant overlap in binding potential across the targets. While Prothrombin yielded the highest number of unique hits (1478), only 30 compounds demonstrated the triple-threat capability of binding F10, F2 and PAR1.

Based only on binding energy, the first docking results indicate that Digoxin and Ledipasvir performed the best. Drug repurposing, however, needs to strike a balance between pharmacokinetic viability and affinity. The comparative ADMET analysis revealed the significant pharmacokinetic hurdles for the selected drugs (Digoxin and Ledipasvir) and confirmed that they were less suitable than the other candidate drugs (Naldemedine and Avodart). The high molecular weight of Digoxin and elevated TPSA contribute to its poor drug-likeness score. These observations clearly indicate the limitations involved in selecting therapeutic usage, but experimental evidence is needed. Similarly, Ledipasvir was observed to have substantial risks for drug–drug interactions, and was hence not considered in this study. Naldemedine remains the primary option among the four selected drugs, followed by Avodart.

The simulation highlights the critical roles of GLU56, GLU65 and GLU66 in stabilizing the ligand Naldemedine within the Coagulation Factor X binding site. The observed stability and interaction occupancy suggest that these residues are essential for drug specificity. The binding of Naldemedine to F10, F2 and PAR1 suggests a therapeutic triple-hit. By disrupting the proteolytic cascade (F10/F2) and the cellular signaling receptor (PAR1), the drug prevents the inflammatory priming of the myocardium. This combined effect potentially raises the threshold for arrhythmia, offering a robust defense against the thrombotic triggers of Sudden Cardiac Arrest (Table 3). These findings align with established antithrombotic targets and suggest that laboratory validations are required for the therapeutic potential of the selected ligand.

The 100 ns Molecular Dynamics simulation provided a rigorous stress test for the Naldemedine–Factor X complex. The stability of the protein backbone and the maintenance of secondary structure elements (alpha-helices and beta-strands) confirm that Naldemedine does not destabilize the target protein. More significantly, a continuing network of interactions with Glu56, Glu65, and Glu66 was highlighted by the MM-GBSA calculations, which confirmed the initial docking energies. Naldemedine is locked into the active site by these amino acids, the acidic residues of which seem to function as a molecular anchor. The binding regions’ low RMSF values support tight binding, indicating that Naldemedine would continue to be effective in physiological blood flow and pressure conditions [24]. The therapeutic combined effect attained by focusing on these proteins’ non-hemostatic functions may be the most important discovery from these findings. Table 3 summarizes the functions of three proteins which extend well beyond the cascade of clotting. They actively contribute to artery irritation [25,26].

A series of pro-inflammatory cytokines are released and macrophage recruitment to the arterial wall is encouraged when Factor X and thrombin activate PAR1. A stable atherosclerotic plaque is placed at risk due to this inflammatory environment. This thrombo-inflammatory connection is crucial in light of the increasing SCA problem in young adults (30–50 years old) [27,28], especially in the Indian population. SCA in these patients is often not the result of a long-term 99% blockage, but rather a sudden rupture of a mildly inflamed plaque that triggers an immediate, fatal electrical arrhythmia. Regarding the clinical implications for sudden cardiac arrest, Naldemedine might target both the clot and the inflammatory tissue by blocking F10, F2, and PAR1. Similar to the combination of low-dose Rivaroxaban and Aspirin, Naldemedine can potentially [29] provide a single-molecule solution to stop thrombin generation while simultaneously preventing platelet receptor activation. However, the study needs more confirmatory laboratory studies. Naldemedine demonstrated some variations from the Lipinski criteria despite its potential, mostly because of its molecular weight and lipophilicity. However, it is a great candidate for further improvement due to its high intestine absorption (79.5%) and absence of hERG inhibition, which means that it does not intrinsically produce arrhythmias. Future studies should focus on in vitro validation to confirm that Naldemedine can reduce cytokine release in PAR1-activated endothelial cells. Furthermore, in vivo models of acute coronary syndrome might reveal whether this triple-target inhibition [30] results in a meaningful decrease in ventricular fibrillation and sudden death.

## 5. Conclusions

The FDA-approved virtual screening of 1657 compounds focused on the protein F10, F2 and PAR1 binding pockets. The virtual screening included four ligands, with Naldemedine, Digoxin, Ledipasvir and Avodart being the most promising potential medications. MD simulation was used to assess the stability of Naldemedine, Digoxin, Ledipasvir and Avodart in the Coagulation Factor X pocket for 100 ns. MD simulation results were consistent with docking data, indicating stability and adequate contact over 100 ns with minor deviations. The study proposes Avodart and Naldemedine as promising candidates for drug repurposing in antithrombotic therapy. This study provides a computational blueprint for the development of next-generation and broad-spectrum anticoagulants. Importantly, these findings are hypothesis-generating and require further in vitro and in vivo validation to confirm the therapeutic potential of the identified leads.

## Figures and Tables

**Table 1:** The binding affinities (kcal/mol) of the selected compounds identified through virtual screening against the triple-target coagulation proteins.

S. No.	Common Elements	Factor X (F10)	Ki (nM)	ΔG	PAR1 (F2R)	Ki (nM)	ΔG	F2 Affinity (kcal/mol)	Ki (nM)	ΔG	Average
1	Naldemedine	−8.361	7.425 × 10^−7^	−8.35424	−7.255	4.80 × 10^−6^	−7.25	−7.803	1.90 × 10^−6^	−7.796668932	−7.806
2	Noxafil	−7.859	1.73 × 10^−6^	−7.85354	−6.779	1.07 × 10^−5^	−6.77	−6.699	1.23 × 10^−5^	−6.693564677	−7.112
3	Dabigatran	−7.348	4.11 × 10^−6^	−7.34133	−6.793	1.05 × 10^−5^	−6.79	−6.507	1.70 × 10^−5^	−6.501720459	−6.883
4	Venetoclax	−7.186	5.40 × 10^−6^	−7.17975	−7.48	3.29 × 10^−6^	−7.47	−6.281	2.49 × 10^−5^	−6.275903827	−6.982
5	Olysio	−7.136	5.87 × 10^−6^	−7.13035	−8.062	1.23 × 10^−6^	−8.06	−7.083	6.42 × 10^−6^	−7.077253113	−7.427
6	Avodart	−7.12	6.03 × 10^−6^	−7.11443	−8.427	6.64 × 10^−7^	−8.42	−6.644	1.35 × 10^−5^	−6.638609302	−7.397
7	Dihydroergotamine	−7.013	7.23 × 10^−6^	−7.00699	−7.833	1.81 × 10^−6^	−7.83	−7.446	3.48 × 10^−6^	−7.439958588	−7.431
8	Rivaroxaban	−6.961	7.89 × 10^−6^	−6.95528	−6.614	1.42 × 10^−5^	−6.61	−6.21	2.80 × 10^−5^	−6.204961434	−6.595
9	Simeprevir Sodium	−6.897	8.79 × 10^−6^	−6.89134	−7.242	4.91 × 10^−6^	−7.24	−6.453	1.86 × 10^−5^	−6.447764272	−6.864
10	Digoxin	−6.84	9.68 × 10^−6^	−6.83425	−6.866	9.26 × 10^−6^	−6.86	−7.081	6.44 × 10^−6^	−7.075254736	−6.929
11	Ledipasvir	−6.8	1.04 × 10^−6^	−6.79178	−6.533	1.62 × 10^−5^	−6.53	−6.084	3.47 × 10^−5^	−6.079063665	−6.472
12	Ltc	−6.585	1.49 × 10^−6^	−6.57895	−6.248	2.63 × 10^−5^	−6.24	−6.102	3.36 × 10^−5^	−6.097049061	−6.312
13	Apixaban	−6.526	1.64 × 10^−6^	−6.52217	−6.736	1.15 × 10^−5^	−6.73	−7.331	4.22 × 10^−6^	−7.325051895	−6.864
14	Trypan Blue	−6.396	2.05 × 10^−5^	−6.39008	−4.725	0.000343712	−4.72	−4.872	0.000268184	−4.868047038	−5.331
15	Glecaprevir	−4.416	0.0005790	−4.41242	−7.421	3.63 × 10^−6^	−7.41	−7.582	2.77 × 10^−6^	−7.575848243	−5.98

**Table 2:** Predicted drug likeness and ADMET analysis of the selected compounds and known compounds.

Property	Naldemedine	Avodart	Digoxin	Ledipasvir	Apixaban	Rivaroxaban
Molecular Weight	570.25	528.22	780.43	888.41	459.19	435.07
Volume	563.94	492.926	767.03	881.162	459.317	392.524
Density	1.011	1.072	1.017	1.008	1.0	1.108
nHA (Hydrogen Bond Acceptors)	10.0	4.0	14.0	14.0	9.0	8.0
nHD (Hydrogen Bond Donors)	4.0	2.0	6.0	4.0	2.0	1.0
nRot (Rotatable Bonds)	7.0	5.0	7.0	16.0	5.0	6.0
nRing (Number of Rings)	8.0	5.0	8.0	10.0	5.0	4.0
MaxRing	6.0	17.0	17.0	13.0	9.0	6.0
nHet (Heteroatoms)	10.0	10.0	14.0	16.0	9.0	10.0
fChar (Formal Charge)	0.0	0.0	0.0	0.0	0.0	0.0
nRig (Rigid Bonds)	37.0	28.0	44.0	50.0	31.0	25.0
Flexibility	0.189	0.179	0.159	0.32	0.161	0.24
Stereo Centers	4.0	7.0	21.0	6.0	0.0	1.0
TPSA	141.18	58.2	203.06	174.64	110.76	88.18
logS	−5.052	−6.16	−3.712	−9.011	−3.681	−3.11
logP	3.617	5.596	1.88	6.511	1.601	1.168
logD	3.245	4.619	2.599	4.303	2.132	1.586
pKa (Acid)	9.45	10.724	6.302	15.534	8.903	7.105
pKa (Base)	7.305	5.223	5.077	5.382	3.612	2.393
Melting point	189.679	210.03	201.996	369.1	181.427	184.427
Boiling point	335.531	380.813	287.666	540.002	320.682	313.074
Medicinal Chemistry						
QED	0.351	0.427	0.162	0.108	0.631	0.779
GASA	1.0	1.0	1.0	1.0	0.0	0.0
Synth (SAscore)	5.0	4.0	6.0	6.0	2.0	2.0
Fsp3	0.469	0.63	0.927	0.469	0.28	0.316
MCE-18	210.0	132.864	150.278	270.389	66.5	74.8
NPscore	0.291	0.739	2.298	−0.209	−1.041	−1.786
Lipinski Rule	0.0	1.0	1.0	1.0	0.0	0.0
Pfizer Rule	0.0	1.0	0.0	0.0	0.0	0.0
GSK Rule	1.0	1.0	1.0	1.0	1.0	1.0
Golden Triangle	1.0	1.0	1.0	1.0	0.0	0.0
PAINS	0 alerts	0 alerts	0 alerts	0 alerts	0 alerts	0 alerts
ALARM NMR	2 alerts	1 alerts	2 alerts	0 alerts	2 alerts	2 alerts
BMS	0 alerts	0 alerts	0 alerts	0 alerts	0 alerts	0 alerts
Chelator Rule	1 alerts	0 alerts	0 alerts	0 alerts	0 alerts	0 alerts
Colloidal aggregators	0.206	0.662	0.74	0.998	0.303	0.275
FLuc inhibitors	0.047	0.009	0.004	0.392	0.257	0.225
Blue fluorescence	0.054	0.064	0.015	0.662	0.413	0.054
Green fluorescence	0.841	0.009	0.0	0.952	0.903	0.864
Reactive compounds	0.001	0.002	0.239	0.0	0.002	0.003
Promiscuous compounds	0.002	0.054	0.74	0.257	0.001	0.0
Absorption						
Caco-2 Permeability	−5.787	−4.776	−5.68	−5.388	−5.781	−5.102
MDCK Permeability	−5.038	−4.762	−5.243	−5.002	−4.958	−4.6
PAMPA	0.0	0.574	1.0	0.0	0.168	0.051
Pgp-inhibitor	0.003	0.678	0.002	1.0	0.002	0.001
Pgp-substrate	0.662	0.135	1.0	0.0	0.0	0.206
HIA (Human Intestinal Absorption)	0.0	0.0	0.001	0.0	0.0	0.0
F_20%_	0.979	0.001	0.162	0.048	0.0	0.0
F_30%_	0.999	0.0	0.02	0.133	0.0	0.0
F_50%_	1.0	0.002	0.923	0.994	0.0	0.0
Distribution						
PPB (Plasma Protein Binding)	94.969	98.306	33.811	98.862	87.944	91.147
VDss	0.207	0.457	−0.642	0.467	0.12	−0.192
BBB (Blood–Brain Barrier)	0.869	0.999	0.0	0.0	0.202	0.0
Fu (Fraction Unbound)	3.942	1.035	54.411	0.827	11.411	7.858
OATP1B1 inhibitor	0.388	0.012	0.926	1.0	0.023	0.002
OATP1B3 inhibitor	0.031	0.039	0.012	1.0	0.113	0.0
BCRP inhibitor	0.182	0.0	0.016	0.001	0.0	0.0
MRP1 inhibitor	0.999	0.767	0.857	0.0	0.837	0.574
Metabolism						
CYP1A2 inhibitor	0.003	0.017	0.0	0.0	0.0	0.803
CYP1A2 substrate	0.025	0.968	0.0	0.0	1.0	0.89
CYP2C19 inhibitor	0.002	0.181	0.0	0.304	0.005	1.0
CYP2C19 substrate	0.001	0.507	0.122	0.001	0.004	0.505
CYP2C9 inhibitor	0.651	0.004	0.0	0.002	0.007	1.0
CYP2C9 substrate	0.0	0.0	0.0	0.0	0.962	0.955
CYP2D6 inhibitor	0.0	0.001	0.0	0.0	0.0	0.162
CYP2D6 substrate	0.35	0.0	0.0	0.0	0.029	0.0
CYP3A4 inhibitor	0.001	0.977	0.0	0.002	0.691	0.999
CYP3A4 substrate	1.0	1.0	0.998	0.013	0.999	1.0
CYP2B6 inhibitor	0.049	1.0	0.82	1.0	0.001	0.968
CYP2B6 substrate	0.0	0.0	0.0	0.0	0.0	0.0
CYP2C8 inhibitor	1.0	0.984	0.218	1.0	0.996	1.0
HLM Stability	0.363	0.016	0.047	0.001	0.086	0.299
Excretion						
_CLplasma_	10.107	7.144	1.413	5.237	3.079	0.594
T_1/2_	1.795	0.657	4.919	0.883	0.918	1.266
Toxicity						
hERG Blockers	0.372	0.59	0.162	0.78	0.817	0.647
hERG Blockers (10 μm)	0.355	0.67	0.864	0.305	0.698	0.369
DILI	0.866	0.433	0.994	0.993	0.965	0.999
AMES Mutagenicity	0.263	0.054	0.979	0.684	0.932	0.759
Rat Oral Acute Toxicity	0.769	0.802	0.999	0.985	0.565	0.214
FDAMDD	0.809	0.998	1.0	0.997	0.858	0.422
Skin Sensitization	0.934	0.279	1.0	0.37	0.003	0.126
Carcinogenicity	0.45	0.263	0.877	0.609	0.972	0.847
Eye Corrosion	0.0	0.0	0.0	0.0	0.0	0.0
Eye Irritation	0.007	0.751	0.0	0.0	0.0	0.016
Respiratory	0.977	0.979	1.0	0.92	0.704	0.485
Human Hepatotoxicity	0.874	0.901	0.438	0.888	0.925	0.881
Drug-induced Nephrotoxicity	0.964	0.913	0.995	0.999	0.874	0.995
Ototoxicity	0.856	0.623	0.982	0.991	0.898	0.699
Hematotoxicity	0.699	0.241	0.947	0.012	0.899	0.352
Genotoxicity	0.995	1.0	1.0	1.0	1.0	1.0
RPMI-8226 (Immunotoxicity)	0.097	0.111	0.965	0.164	0.213	0.079
A549 Cytotoxicity	0.46	0.29	0.977	0.498	0.145	0.034
Hek293 Cytotoxicity	0.663	0.95	0.994	0.976	0.935	0.412
Drug-induced Neurotoxicity	0.131	0.564	0.014	0.989	0.992	0.966
Bioconcentration Factors	1.073	2.354	0.518	1.544	0.086	0.147
IGC_50_	4.216	3.747	3.217	5.139	3.096	2.806
LC_50_FM	4.907	4.926	3.838	7.101	3.588	3.381
LC_50_DM	5.122	5.673	4.739	7.593	4.431	4.424
Tox21 pathway						
NR-AhR	0.71	0.014	0.001	0.0	0.381	0.004
NR-AR	0.078	0.984	0.741	0.0	0.318	0.028
NR-AR-LBD	0.042	0.289	0.999	0.0	0.018	0.011
NR-Aromatase	0.013	0.011	0.998	0.616	0.202	0.05
NR-ER	0.931	0.755	0.995	0.999	0.399	0.933
NR-ER-LBD	0.001	0.005	0.351	0.172	0.028	0.068
NR-PPAR-gamma	0.704	0.0	0.056	0.0	0.017	0.002
SR-ARE	0.539	0.816	0.169	0.997	0.486	0.068
SR-ATAD5	0.959	0.001	0.907	0.478	0.046	0.008
SR-HSE	0.011	0.001	0.04	0.003	0.007	0.005
SR-MMP	0.079	0.139	0.989	0.989	0.159	0.0
SR-p53	0.047	0.045	0.999	0.931	0.486	0.214
Toxicophore rules						
Acute Toxicity Rule	0	0	0	0	0	1 alerts
Genotoxic Carcinogenicity Rule	4 alerts	1 alerts	2 alerts	4 alerts	1 alerts	2 alerts
Non-Genotoxic Carcinogenicity Rule	2 alerts	1 alerts	1 alerts	2 alerts	0	0
Skin Sensitization Rule	6 alerts	6 alerts	4 alerts	2 alerts	1 alerts	1 alerts
Aquatic Toxicity Rule	3 alerts	5 alerts	5 alerts	3 alerts	0	2 alerts
Non-Biodegradable Rule	0	1 alerts	1 alerts	2 alerts	0	2 alerts
SureChEMBL Rule	0	1 alerts	0	0	0	0
FAF-Drugs4 Rule	2 alerts	2 alerts	1 alerts	3 alerts	1 alerts	2 alerts

**Table 3:** Multi-target roles and impact of Naldemedine.

Target Protein	Classical Hemostatic Role	Non-Hemostatic (Inflammatory) Role	Impact of Naldemedine Inhibition (Based on Results)
Factor X (F10)	Converts Prothrombin into Thrombin.	Triggers pro-inflammatory cytokines via PAR activation; promotes cell migration.	MD simulations (100 ns) confirm exceptional stability via GLU56/GLU66 anchors.
Prothrombin (F2)	Generates Thrombin, the final enzyme for fibrin clots.	Drives plaque rupture and activates immune cells (macrophages) in vessel walls.	Highest binding affinity can prevent the Thrombin surge that destabilizes cardiovascular health.
PAR1 (F2R)	Mediates platelet aggregation in response to Thrombin.	Acts as a master switch for vascular leakage and chronic inflammation.	Blocks signaling ADMET, confirms no BBB crossover, ensuring localized vascular protection.

## Data Availability

The original contributions presented in the study are included in the article/Supplementary Materials. Further inquiries can be directed to the corresponding author.

## Supplementary Materials

The following supporting information can be downloaded at https://www.mdpi.com/article/10.3390/biomedicines14051120/s1. Figure S1: Top panel: Alpha fold and crystal structure comparison docking. Bottom panel: Validation in silico docking using Factor Xa with known inhibitors (Apixaban and Rivaroxaban) using both PDB and AlphaFold structures. Figure S2. Structural validation of Coagulation Factor X (F10), Proteinase-activated receptor 1 (PAR1) and Prothrombin. Left: Representative 3D structures of F10, PAR1 and Prothrombin are displayed as cartoon models colored by pLDDT scores. The color gradient indicates model confidence. Blue represents very high confidence (pLDDT > 90) while orange and red represent low to very low confidence (pLDDT < 50). Right: pLDDT score plots illustrating the per-residue confidence levels across the protein sequences for five ranked structural models. Highconfidence regions (blue) generally correspond to well-defined folded domains, whereas low-confidence regions (red/orange) correspond to intrinsically disordered or flexible loops.
