# Supplementary material for: Multi-Target Inhibition of F10/F2/PAR1 Through In Silico Drug Repurposing of Avodart and Naldemedine to Prevent Thrombotic-Induced Sudden Cardiac Arrest
Source: Biomedicines. 2026 May 15;14(5):1120. doi: 10.3390/biomedicines14051120 (PMC13204583; doi:10.3390/biomedicines14051120)
Supplement: Supplementary file 1 [file biomedicines-14-01120-s001.zip › biomedicines-4179172-supplementary.pdf]

Multi-Target Inhibition of F10/F2/PAR1 Through In Silico Drug Repurposing of Avodart and Naldemedine to Prevent Throm-botic-Induced Sudden Cardiac Arrest

Abeer M. Al-Subaie and Sayed AbdulAzeez

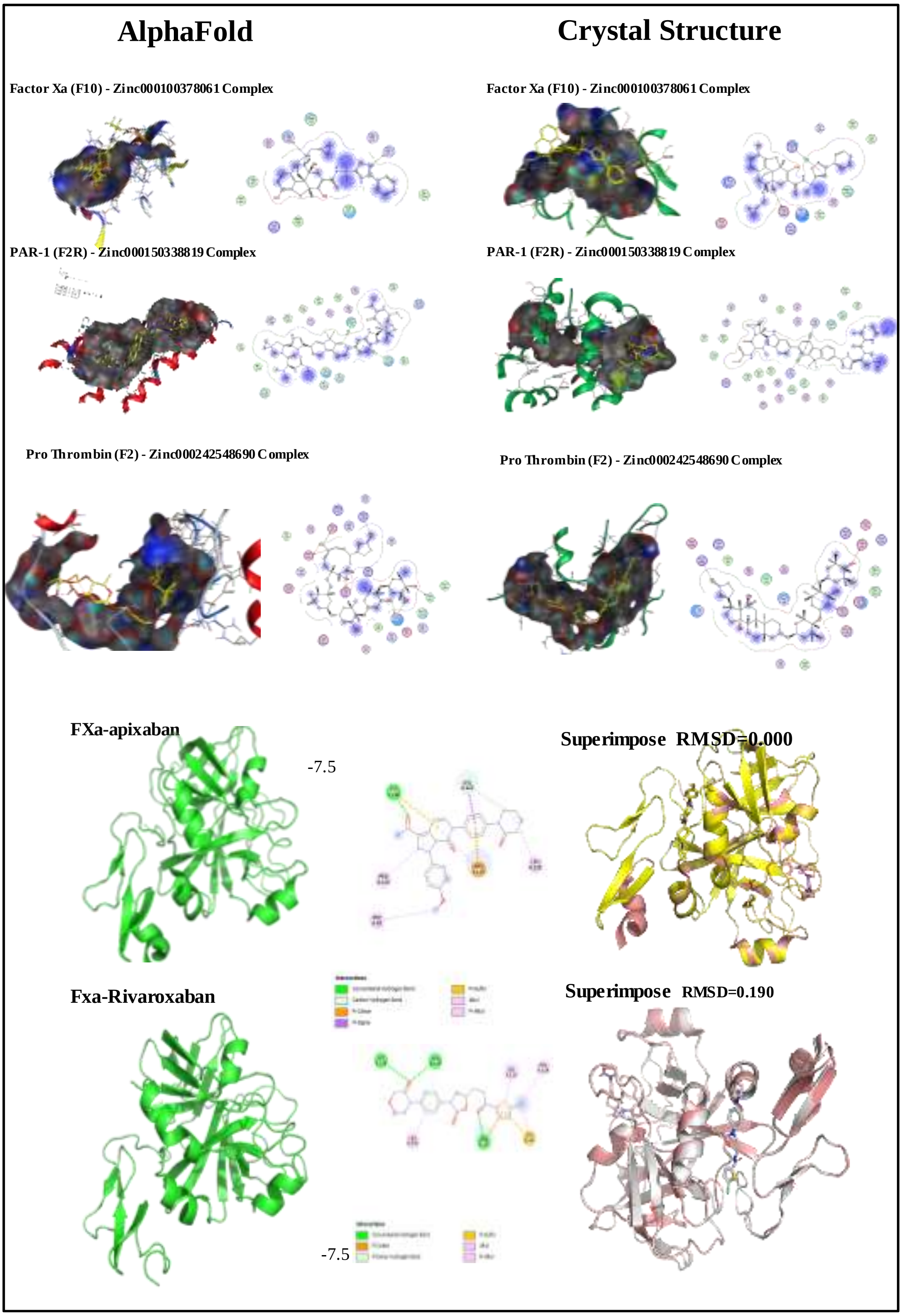

Figure S1: Top panel: Alpha fold and crystal structure comparison docking. Bottom panel: Validation in silico docking using Factor Xa with known inhibitors (Apixaban and Rivaroxaban) using both PDB and AlphaFold structures.

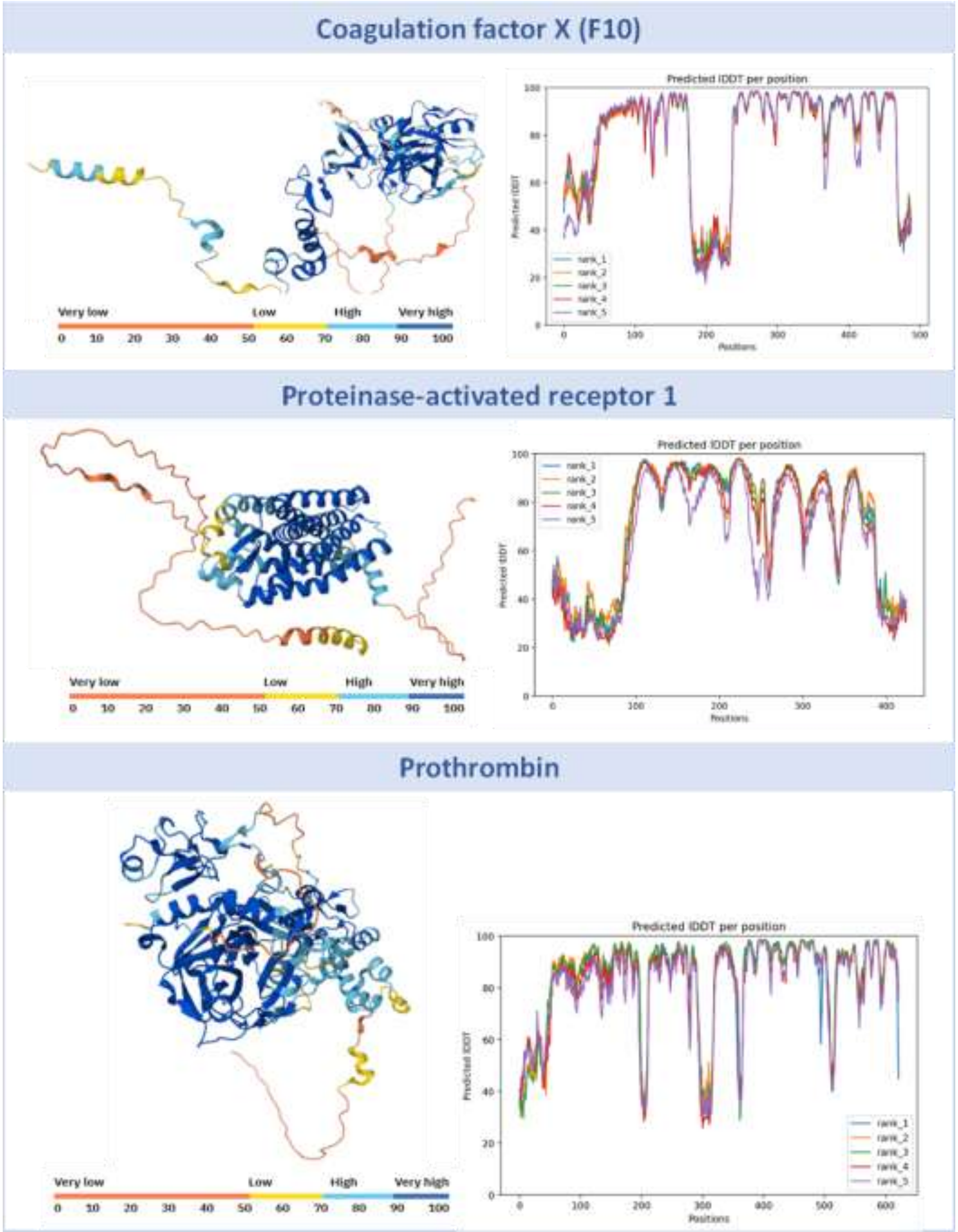

Figure S2. Structural validation of Coagulation Factor X (F10), Proteinase-activated receptor 1 (PAR1) and Prothrombin. Left: Representative 3D structures of F10, PAR1 and Prothrombin are displayed as cartoon models colored by pLDDT scores. The color gradient indicates model confidence. Blue represents very high confidence (pLDDT > 90) while orange and red represent low to very low confidence (pLDDT < 50). Right: pLDDT score plots

illustrating the per-residue confidence levels across the protein sequences for five ranked structural models. High-confidence regions (blue) generally correspond to well-defined folded domains, whereas low-confidence regions (red/orange) correspond to intrinsically disordered or flexible loops.
